# Heterogeneous Surface CD79b Expression in Aggressive B-Cell Lymphomas Assessed by Flow Cytometry on Lymph Node Biopsies

**DOI:** 10.3390/cancers16233968

**Published:** 2024-11-26

**Authors:** Elena Maiolo, Silvia Bellesi, Fabrizia Campana, Camilla Iacovelli, Rosalia Malafronte, Gabriele Schiaffini, Eleonora Alma, Flaminia Bellisario, Marcello Viscovo, Simone D’Innocenzo, Alessia Toscano, Francesco D’Alò, Valerio De Stefano, Luigi Maria Larocca, Stefan Hohaus

**Affiliations:** 1 Dipartimento di Scienze di Laboratorio ed Ematologiche, Fondazione Policlinico Universitario A. Gemelli IRCCS, 00168 Rome, Italy; elena.maiolo@policlinicogemelli.it (E.M.); camillaiacovelli@gmail.com (C.I.); malafronte.rosalia@gmail.com (R.M.); eleonora.alma@policlinicogemelli.it (E.A.); flaminia.bellisario@guest.policlinicogemelli.it (F.B.); simone.dinnocenzo@policlinicogemelli.it (S.D.); alessia.toscano@policlinicogemelli.it (A.T.); francesco.dalo@unicatt.it (F.D.); valerio.destefano@unicatt.it (V.D.S.); stefan.hohaus@unicatt.it (S.H.); 2 Sezione di Ematologia, Dipartimento di Scienze Radiologiche ed Ematologiche, Università Cattolica del Sacro Cuore Facoltà di Medicina e Chirurgia, 00168 Rome, Italy; fabrizia.campana@gmail.com (F.C.); gab.schiaffini@gmail.com (G.S.); viscovomarcello@gmail.com (M.V.); 3 Patologia Oncoematologica, Dipartimento di Scienze della Salute della Donna, del Bambino e di Sanità Pubblica, Fondazione Policlinico Universitario A. Gemelli IRCCS, 00168 Rome, Italy; luigimaria.larocca@policlinicogemelli.it

**Keywords:** aggressive B-cell lymphoma, CD79b, flow cytometry, target therapy, lymph node biopsies

## Abstract

**Background:** CD79b is a B-cell-specific antigen that is crucial to the B-cell receptor and is considered a key target for treatment in aggressive B-cell lymphomas. **Methods:** While immunohistochemical studies have shown widespread expression of CD79b in mature B-cell-derived lymphomas, flow cytometry allows for precise measurement and differentiation between surface and intracellular localization. **Results:** In our comparative analysis, we discovered that CD79b expression percentages and mean fluorescence intensity (MFI) were lower in a group of 127 cases of aggressive B-cell lymphomas compared to a control group of benign reactive hyperplasia. We also observed significant variability in the surface expression of CD79b among lymphoma cases, with 18% showing predominantly intracellular positivity. There was a strong correlation between the surface expression of CD79b and clonal light chains. Notably, primary mediastinal B-cell lymphomas exhibited significantly lower surface CD79b expression compared to other lymphoma subtypes (median 0.8% IQR 0–48.5 vs. 80% IQR 24–97, *p* = 0.0005). Furthermore, patients over 60 years old and those with a higher Revised International Prognostic Index (R-IPI) had significantly higher CD79b expression, both of which are associated with a significant benefit from adding an anti-CD79b drug conjugate to first-line chemotherapy in diffuse large B-cell lymphomas. **Conclusions:** In conclusion, the quantitative flow cytometric analysis of CD79b surface expression in aggressive B-cell lymphomas provides clinically relevant information, highlighting its potential usefulness in guiding therapeutic decisions.

## Simple Summary

CD79b, a B-cell-specific antigen, is highly expressed in mature B-cell lymphomas, making it a promising therapeutic target. This study employs flow cytometry to assess CD79b in lymph node biopsies from 127 cases of large B-cell lymphomas (LBCLs), compared to benign reactive hyperplasia. We found that LBCLs exhibited lower and heterogeneous CD79b expression, with 18% of cases showing almost exclusively intracellular positivity and, in particular, low surface expression in primary mediastinal B-cell lymphomas. A positive correlation was observed between CD79b expression and surface immunoglobulin light chains. Patients over 60 years old and those with high-risk disease (Revised International Prognostic Index of 3–5) demonstrated higher surface CD79b expression. These findings align with results from the POLARIX study, where the addition of a CD79b-ADC (antibody–drug conjugate) to first-line therapy improved outcomes for patients with similar characteristics. Flow cytometry on tissue biopsies may offer valuable insights into target antigen expression, including CD79b.

## 1. Introduction

The B-cell receptor (BCR) is a complex hetero-oligomeric structure wherein ligand binding and signal transduction domains are compartmentalized within distinct receptor subunits. These subunits consist of surface immunoglobulin (sIg) and a non-covalently associated complex of two proteins, Igα and Igβ, designated CD79a and CD79b [1]. The specific binding of antigens takes place through the cell surface immunoglobulin segment of the BCR, with CD79a and CD79b playing pivotal roles in endocytosis and signal transduction [2]. This interplay is a critical pathway for the activation of B cells. The initiation of the signal transduction cascade results in the internalization of the complex, including CD79b, and the subsequent trafficking of these molecules to late endosomes, ultimately contributing to antigen presentation [3].

CD79b exhibits a nearly exclusive expression pattern on B cells and B-cell neoplasms. During B-cell development, CD79b initially appears on the cell surface at the pre-B-cell stage in conjunction with the heavy Ig chain. This antigen persists throughout all stages of B-cell differentiation, undergoing downregulation just before differentiation into plasma cells. Notably, cytoplasmic expression of CD79b in the early stages precedes the surface expression of both CD79b and the Ig heavy chain.

Unlike the prevalent expression of CD79a, CD79b is typically absent in pre-B-acute lymphoblastic leukemia (ALL), plasma cell tumors, and chronic lymphocytic leukemia (CLL) cases. In contrast, CD79b finds widespread expression in mature B-cell neoplasms, encompassing 80–90% of cases [4,5].

Limited data currently exist regarding the evaluation of CD79b expression through immunohistochemistry. Pfeifer et al. conducted immunohistochemical (IHC) staining for CD79b cell-surface expression and found strong and widespread expression of CD79b in 28 cases of diffuse large B-cell lymphoma (DLBCL) [6]. Tilly et al. used staining intensity and the number of positively stained tumor cells to establish an H-score, ranging from 0 to 300 [7]. In their study, all 38 samples showed positive CD79b expression, with H-scores varying from 35 to 295. Naoi et al. analyzed 576 cases of de novo DLBCL and identified different IHC CD79b staining patterns, with the majority being cytoplasmic (75%), membranous (14.9%), and 9% negative cases. These patterns significantly impacted H-scores, with the highest scores associated with the membranous pattern and significant variation across the cell of origin (COO) [8]. Flow cytometry (FC) has become a valuable technique for quantifying CD79b levels on B-cell lymphoma cells in peripheral blood or bone marrow. High CD79b expression has been observed in mantle cell lymphoma, hairy cell leukemia, and splenic marginal zone lymphoma, while this antigen was found to be positive only in rare cases of chronic lymphocytic leukemia (CLL) [9,10]. Majzner et al. analyzed CD79b expression in 18 cases of DLBCL using FC, revealing significant interpatient heterogeneity [11]. Furthermore, CD79b expression showed a positive correlation with other B-cell markers, including CD20 and surface immunoglobulin (sIg) [12]. The nearly exclusive expression of CD79b on B cells and B-cell lymphomas, coupled with its internalization upon antigen binding, has sparked a growing interest in CD79b as a therapeutic target for antibody–drug conjugates (ADCs). Polatuzumab Vedotin (PV) is a humanized immunoglobulin (IgG1) monoclonal antibody designed to target CD79b, delivering monomethyl auristatin (MMAE), a potent microtubule inhibitor [13]. In combination with Rituximab-Bendamustine, PV has been approved for the treatment of relapsed/refractory (R/R) DLBCL. This therapeutic combination has demonstrated a significantly higher complete response (CR) rate and reduced the risk of death by 58% compared to Rituximab-Bendamustine in patients (pts) with R/R DLBCL ineligible for transplantation [13,14]. A recent analysis of the Polarix study underscores the positive impact of adding PV to standard first-line therapy, demonstrating improved progression-free survival (PFS) in pts with DLBCL. This effect is particularly pronounced in high-risk cases, with an International Prognostic Index (IPI) of 3–5 and activated B-cell-like (ABC) cell of origin subtype [7,15].

The identification of CD79b as a target for therapeutic intervention and the limited available data on its expression in DLBCL have prompted our investigation. The primary objective of our study was to quantitatively evaluate the surface expression of CD79b in lymph node biopsies obtained from pts with aggressive B-cell lymphomas, employing FC as an analytical tool.

## 2. Materials and Methods

### 2.1. Patients and Samples

We conducted an analysis of lymph node samples obtained from December 2015 to January 2024, encompassing 127 consecutive pts diagnosed with aggressive B-cell lymphomas. Among these, 101 cases were collected at the primary diagnosis, and 26 cases were obtained at relapse. The median age of our patient cohort was 65 years, with an age range spanning from 19 to 88 years. The gender distribution included 63 male and 64 female. Histological diagnoses included 89 cases of diffuse large B-cell lymphoma not otherwise specified (NOS), 2 cases of T cell/histiocyte-rich B-cell lymphoma, 11 cases of transformed follicular lymphoma, 13 cases of high-grade B-cell lymphoma, and 12 cases of PMBCL. For DLBCL NOS, the COO was determined using the Hans algorithm [16], categorizing 33 cases as germinal-center B-cell (GCB), 48 as non-GCB, and 8 cases as not evaluable. Table 1 summarizes the pts’ characteristics. Additionally, we included a control group consisting of 16 lymph node biopsies diagnosed immunohistochemically as benign reactive hyperplasia (BRH) for comparative analysis. This study was conducted in adherence to the Helsinki criteria and received approval from the local ethics committee as part of the project PNRR MAD-2022-12376707 (ID 5444 Prot 003275/23). Pts provided informed, signed consent for the biobanking of samples and the anonymized use of their data in this study.

### 2.2. Flow Cytometry

Multiparametric eight-color flow cytometric immunophenotyping was carried out as follows.

To generate cell suspensions from tissue material, we performed a manual or mechanical disaggregation as described by Vallangeon BD et al. [17].

Prior to staining, the cellular suspensions underwent two washes with phosphate-buffered saline (PBS) supplemented with 1% bovine serum albumin (BSA), after which they were suspended in 2 mL PBS. The leucocyte counts of these cellular suspensions were determined using the ADVIA2120 instrument, guiding the calculation of the final staining volume. Between 5 × 10^5^ and 1 × 10^6^ cells were stained with a standard panel of directly conjugated antibodies to eight surface markers, including the main diagnostic antigens of B-cell lymphomas: Kappa-V450 (clone TB28-2 Becton Dickinson BD, Franklin Lakes, NJ, USA), CD45-V500 (clone 2D1 BD), CD20-FITC (clone B9E9 Beckman Coulter BC), CD79b-PE clone (CB3-1 BC), CD5-PerCP-Cy5.5 (clone L17F12 BD), CD19-PE-Cy7 (clone J3-119 BC), CD10-APC (clone HI10a BD), and Lambda-APC-H7 (clone 1-155-2 BD). Specifically, the CD79b antigen was identified using the CB3-1 antibody, which targets an extracellular epitope of CD79b but is also detectable in the cytoplasm of surface immunoglobulin (sIg) negative B-cell precursors, particularly in pre-B cells and 80% of pro-B cells [18]. Staining was carried out for 20 min at room temperature, followed by the lysis of red blood cells (RBCs) in the samples using 2 mL of FACSLyse (Becton Dickinson) for 10 min at room temperature. Subsequently, the cells were pelleted, washed, and resuspended in PBS plus 1% BSA for further analysis. For intracytoplasmic detection of CD79b and light chains, a specific intracytoplasmic staining procedure was implemented using IntraPrep Permeabilization reagent (Beckman Coulter). Data were acquired with BDFACSCanto for the first 43 consecutive pts and with DXFlex (Beckman Coulter) for the subsequent 84 pts and for all 16 BRH cases. Data were analyzed using BDFACSDiva Software (version 2.0, Becton Dickinson) and CyExpert Software (version 9.2, Beckman Coulter, Brea, CA, USA). Instrument precision was maintained by daily calibration to standardized beads.

After the exclusion of cell debris and doublets using physical parameters, CD19+ events were gated as the population of interest. The identification of an aberrant CD19+ B-cell population suggestive of aggressive B-cell lymphoma was based on the detection of surface or cytoplasmic immunoglobulin light chain clonal restriction or on the absence of surface and/or cytoplasmic light chain expression on otherwise mature CD19+ CD20+ CD45+ (high-expression) B cells [19]. This was coupled with an observation of increased forward scatter (FSC-A) and side scatter (SSC-A) physical parameters [20,21,22,23]. The cluster of CD5+ T-cells was used as an internal negative control for background staining.

Two different modalities were employed to characterize the expression levels of the surface markers of interest (sKappa, sLambda, CD20, CD10, CD5, and CD79b): the percentage of positively stained cells with respect to the totality of pathological B cells and the median fluorescence intensity (MFI). The definition of surface antigen expression was as follows: strongly positive if expressed on >70% of total pathological CD19+ B cells, partially positive when expressed on 20–69%, weakly positive if expressed on 1–19%, and barely positive/negative if less than 1% of positive events were observed within pathological CD19+ B-cell cluster. Recognizing the variability in MFI values due to instrumental settings, we analyzed samples separately according to each cytometer. Furthermore, the relative median fluorescence intensity (RMFI) was calculated as the ratio between the MFI of each surface antigen on the pathological CD19+ population and on CD5+CD19− T cells in the same sample. This dual-parameter assessment, as percentage and R-MFI, provided a comprehensive and nuanced characterization of the expression patterns.

### 2.3. Statistical Analysis

Statistical comparisons for differences in categorical variables between two groups were conducted using the Fisher test, while differences in continuous variables were assessed using the Mann–Whitney test or Kruskal–Wallis one-way ANOVA (using the Tukey–Kramer test for post hoc analysis). Data were summarized as medians and interquartile ranges (IQR). Spearman correlation analysis was employed to examine associations between parametric continuous variables. The threshold for statistical significance was set at *p* < 0.05. All statistical analyses were performed using the NCSS10 Software (version 10.0) and GraphPad Prism (version 10.2.0).

## 3. Results

In our study focusing on CD79b expression, we examined 127 lymph node samples from pts with histological diagnosis of aggressive B-cell lymphomas; furthermore, we included 16 cases of benign reactive hyperplasia (BRH) as controls. An aberrant mature B-cell cluster was exclusively observed in lymphoma cases. In particular, 90% of all cases (114/127) showed clonal surface light chain restriction (66 kappa, 48 lambda). The remaining 10% (13/127) exhibited an otherwise mature CD19+ CD20+ CD45+ B-cell population lacking surface light chain expression and presenting high FSC-A and SSC-A; notably, 54% of these cases (7/13) were diagnosed as PMBCL.

**Figure 1:**
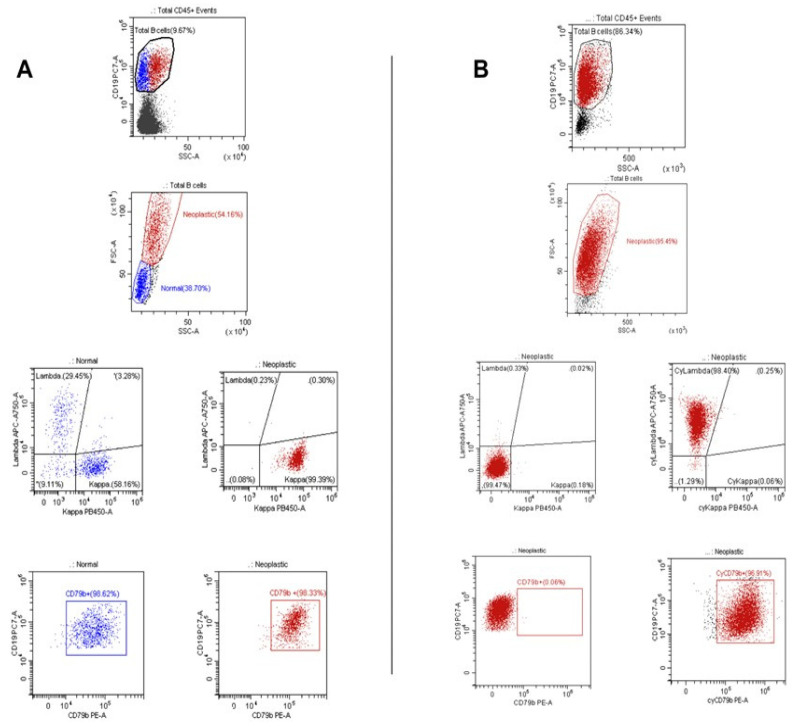
Distinct CD79b expression patterns among lymphoma cases. (**A**) Strongly positive surface CD79b expression. In the contest of CD19+ B cells, we can identify two distinct populations (first plot) classified as normal or neoplastic using physical parameters (second plot). Normal B cells (in blue) show a typical polyclonal distribution of surface light chains and strong CD79b expression. Also, the neoplastic population (in red) displays a strong CD79b positivity but shows clonal restriction for the kappa light chain. (**B**) Intracytoplasmic staining in a patient without surface CD79b and light chain expression. The entire CD19+ population in the sample is composed of neoplastic B cells (in red), identified by physical parameters (first plot). Normal B cells are absent (second plot). Surface staining for CD79b and Ig on the neoplastic population is negative (left plots), but both markers are highly present in the cytoplasm (plots on the right).

In 98.4% (125/127) of biopsies, the pathological B-cell population expressed CD20 strongly (median value 98%, IQR 96–99). CD10 was expressed in 42.5% of cases (54/127), with a median value of 95% (IQR 88–99), featuring strong positivity in 34.6% (44/127) and partial positivity in 7.9% (10/127). CD5 was strongly expressed in 13.4% (17/127) of cases, with a median value of 97.5% (IQR 84.5–99). The median percentage of CD79b expression on pathological B cells was 75% (IQR 10–97). CD79b was strongly positive (>70% of pathological cells being positive) in 53.5% of biopsies (68/127) (Figure 1A), partially positive (20–69% of pathological cells being positive) in 19.6% (25/127), weakly positive (1–19% of pathological cells being positive) in 8.7% (11/127), and barely positive/negative (<1% of pathological cells being positive) in 18.1% (23/127) of cases. Notably, in all lymphoma cases, CD79a expression was detected by IHC staining.

In 18/127 (14%) of DLBCL cases, a significant cluster of normal residual B cells with a polyclonal light chain expression was observed. These events were always positive for surface CD79b expression with a median value of 85% (IQR 58–98). Furthermore, in three pts who were negative for surface CD79b expression on pathological B cells, a positive CD79b expression was observed in residual normal B cells in the same sample as internal staining control.

We extended our analysis to investigate the intracellular expression of CD79b in 9/23 pts with negative surface staining for this antigen. In all cases, we consistently observed a high level of intracellular CD79b expression (median percentage of expression 97%, IQR 95–99). Additionally, among these nine pts, five lacked surface expression of immunoglobulin light chains. Intriguingly, we identified intracellular clonal restriction for light chains in all five cases, with three exhibiting Cykappa and two displaying Cylambda clonality (Figure 1B). This finding provides further insight into the phenotypic characteristics of cases with negative surface CD79b staining, emphasizing the presence of intracellular CD79b expression.

**Figure 2:**
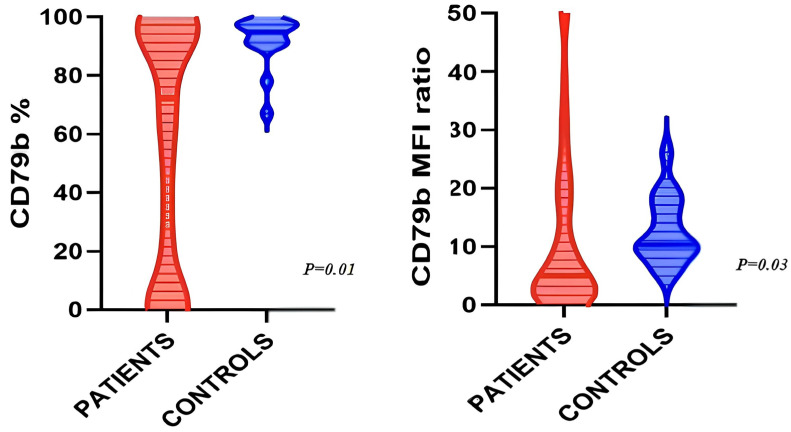
Differences in CD79b expression between large B-cell lymphoma cases and controls. Benign reactive hyperplasia (BRH) polyclonal B cells exhibit a higher percentage of CD79b expression compared to neoplastic B cells (median 94.7%, IQR 90–98 vs. 72% IQR 3–97 respectively, *p* = 0.01). Additionally, a higher CD79b-RMFI was noted in BRH compared to lymphoma cases (median 10.35, IQR 9.1–17.2 vs. 5.05, IQR 1.85–18.5, *p* = 0.03).

Comparing CD79b expression between 16 BRH cases and 84 lymphoma cases, both analyzed with the DXFlex cytometer, we observed a CD79b strong expression (higher than 70%) in 97.5% of polyclonal B cells in BRH. Moreover, BRH polyclonal B cells displayed a higher percentage of CD79b expression compared to LBCL pathological B cells (median 94.7% IQR 90.5–97.5 vs. 72% IQR 3–97, respectively, *p* = 0.01). Also, a higher CD79b-RMFI was observed in BRH compared to LBCL cases (median 10.35, IQR 9.1–17.2 vs. 5.05, IQR 1.85–18.5, *p* = 0.03) (Figure 2).

**Figure 3:**
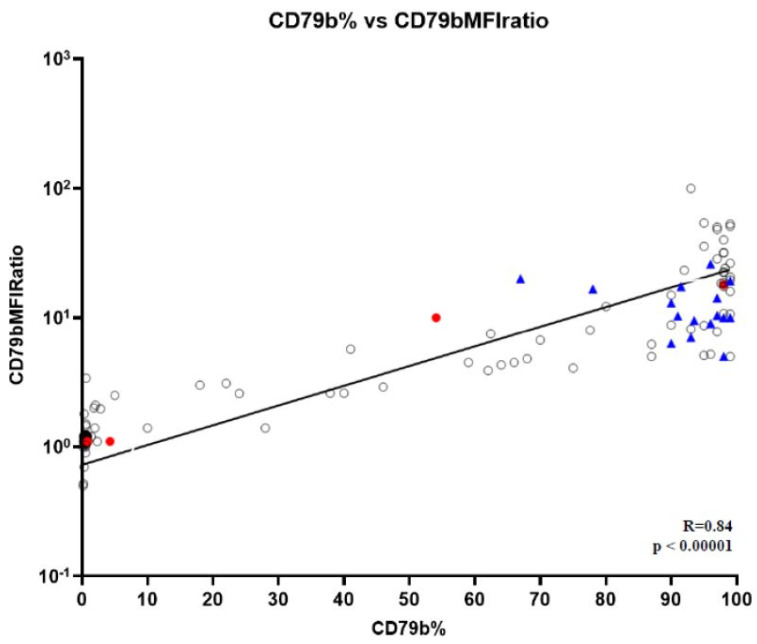
Correlation between CD79b percentage and RMFI in neoplastic B cells and controls. Logarithmic positive correlation between CD79b expression percentage and RMFI in the entire group of neoplastic B cells and benign reactive hyperplasia (BRH) (R = 0.84, *p* < 0.00001). Black circles represent all pts with large B-cell lymphomas. Red circles represent PMBCL. Blue triangles indicate BRH cases.

A robust positive correlation was observed between CD79b percentage of expression and CD79bRMFI considering the entire cohort of DLBCL and BRH (R = 0.84, *p* < 0.00001) (Figure 3).

**Figure 4:**
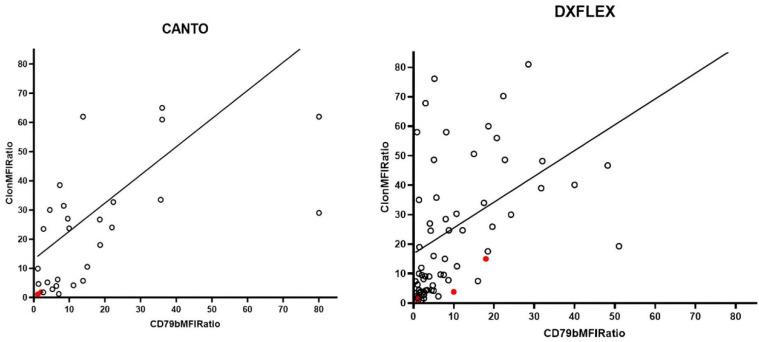
Correlation between CD79b and light chain R-MFI using both CANTO and DXFLEX instruments. A strong positive correlation was observed between CD79b-RMFI and clonal light-chain RMFI regardless of the instrument used for the analysis (Spearman’s R = 0.77, *p* = 0.005 for BD Canto, R = 0.65, *p* < 0.0001 for DxFlex). Red circles represent PMBCL, and black circles represent all other lymphoma cases.

We proceeded to explore associations between CD79b and the expression of other surface markers, conducting a separate analysis for pts analyzed with DxFlex and BD Canto due to a trend of different CD79b RMFI between the two instruments (median 9.7, IQR 4.15–22.1 for Canto, and median 7.25, IQR 2.5–18 for DxFlex, *p* = 0.09). Irrespective of the instrument used, a robust correlation emerged between CD79b-RMFI and clonal light-chain RMFI (Spearman R = 0.77, *p* = 0.005 for BD Canto, and R = 0.65, *p* < 0.0001 for DxFlex) (Figure 4).

Particularly, clonal light-chain RMFI was significantly lower in pts with CD79b < 20% compared to others (median 2.63, IQR 1.16–4.23 versus 23.85, IQR 5.7–33.5, *p* = 0.00056 for BD Canto; median 4.4, IQR 1.5–9.8 versus 25.9, IQR 8.4–49.6, *p* < 0.00001 for DxFlex). No variations in CD79b expression were observed considering expression levels of other B-cell lymphoma markers, such as CD10, CD5, CD19, and CD20.

In our examination of the different histological subtypes of aggressive B-cell lymphomas, PMBCL stood out with significantly lower surface CD79b expression compared to others (median 0.8% IQR 0–48.5 vs. 80% IQR 24–97, respectively, *p* = 0.0005). Instead, we observed no significant variations in CD79b levels based on COO subtypes GC and non-GC (median for GC 90% IQR 49.5–97 vs. 63.5% for non-GC IQR 2.33–96, *p* = 0.11).

In our examination of the differences between 101 samples obtained at first diagnosis and 26 cases at relapse, there was no statistically significant difference in the distribution of CD79b positivity (median 80%, IQR 21.5–97 at diagnosis vs. 64%, IQR 2.8–90 at relapse, *p* = 0.18). Specifically, the number of pts with CD79b expression < 1% did not differ between disease onset (20/101, 19.8%) and relapse (6/26, 23.1%) (*p* = 0.7), despite no patient at relapse having received anti-CD79b therapy as part of first-line treatment.

**Figure 5:**
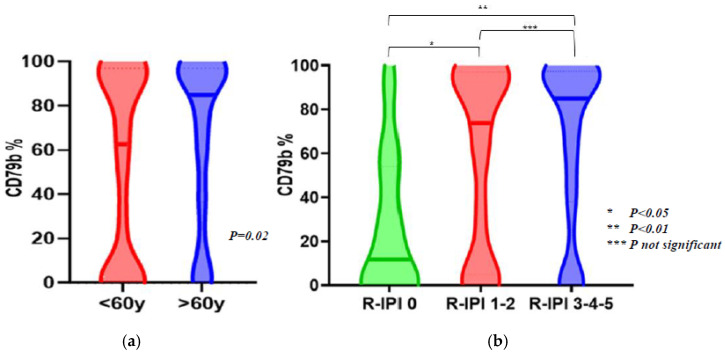
Correlation between CD79b expression and clinical parameters. The figures show significantly higher levels of CD79b expression in pts older than 60 years ((**a**) *p* = 0.02) and significantly lower levels in pts with low R-IPI score ((**b**) R-IPI 0 vs. R-IPI 1-2, *p* = 0.04; R-IPI 0 vs. R-IPI 3-4-5, *p* = 0.009). These clinical parameters are both associated with better response to Polatuzumab-Vedotin-containing regimens.

Analyzing associations of CD79b with clinical characteristics, we observed a significantly higher percentage of CD79b expression in pts older than 60 years (median 56.5% IQR 0.5–90 in the group < 60 years vs. 85% IQR 36.5–97 in pts > 60 years, *p* = 0.02) (Figure 5a) and in pts with higher Ann Arbor stage (median 53.5% IQR 4.2–68 in Ann Arbor stage I–II group vs. 90% IQR 74–95 in Ann Arbor stage III–IV, *p* = 0.02). No statistically significant associations were found for other factors constituting IPI score (serum LDH levels, extranodal sites, and ECOG). Examining CD79b expression among different prognostic subgroups according to R-IPI classification, we found it lower in pts with R-IPI 0 compared with others (*p* = 0.03; median 11.7%, IQR 0.2–51 for R-IPI 0, median 74%, IQR 7.5–97 for R-IPI 1-2, and median 85%, IQR 37.95–97 for R-IPI 3–5) (Figure 5b). A trend of major CD79b expression was seen in multivariate analysis for age > 60 (*p* = 0.07).

## 4. Discussion

CD79b, a B-cell-specific antigen recently targeted for therapy in aggressive B-cell lymphomas, exhibits widespread expression on mature B-cell-derived lymphomas, as evidenced by immunohistochemical studies. Given the limitations in quantifying expression and distinguishing between surface and intracellular localization through immunohistochemistry (IHC), FC emerges as a more precise and quantitative method to estimate antigen expression and compartmentalization. Within our cohort of 127 cases, we noted heterogeneous surface expression of CD79b among aggressive B-cell lymphomas, with approximately 20% of cases displaying nearly exclusive intracellular positivity. This observation is consistent with earlier studies that highlight significant interpatient variability in CD79b expression in FC analysis of de novo DLBCL samples [11]. Both the percentage of CD79b expression and CD79b-MFI were found to be lower in aggressive B-cell lymphoma cases compared to a control group of BRH. Additionally, our observations revealed a correlation between the surface and intracellular expression patterns of CD79b and immunoglobulin light chains. This correlation is unsurprising given that CD79b associates with immunoglobulin molecules to form the BCR, with cycling between the cell surface and intracellular compartments being intrinsic to their physiological function. Existing literature supports the idea that CD79b endocytosis is linked to the simultaneous internalization of the entire BCR complex and that CD79b internalization does not occur as a monomer [24,25]. BCR endocytosis serves as the gateway for BCR-bound antigens to traverse to lysosomes for antigen processing. This orchestrated process enables subsequent presentation by B cells, playing a pivotal role in recruiting T cell help.

Our investigation revealed that PMBCL cases exhibited notably low surface expression levels of CD79b and often lacked surface immunoglobulin light chain expression. This observation aligns with previous reports indicating the absence of surface immunoglobulin (Ig) in 50–60% of PMBCL cases [20,26].

Given that CD79b plays a crucial role in mediating signaling through the BCR upon antigen binding, we can only speculate on the functional consequences of the low or absent expression of CD79b and its potential impact on the alteration of the signaling pathway in lymphoma cells. Similar scenarios have been explored in CLL B cells, where the absence of surface CD79b and low surface light chain levels are typical phenotypic characteristics. Intriguingly, a distinct subpopulation of CLL cells has been identified, exhibiting reduced BCR signaling and an increased propensity for independent internalization of surface immunoglobulin M (sIgM) and CD79b [27]. These data highlight changes in internalization dynamics in B-cell malignancies.

In CLL, an alternative splicing event resulting in a truncated form of CD79b, which lacks exon 3 that encodes the extracellular Ig-like domain, has been described [28]. This mechanism could potentially account for reduced BCR expression on the surface of B-CLL cells. It has to be explored whether this mechanism could also contribute to reduced CD79b surface expression in aggressive B-cell lymphomas. Understanding the dependency of different lymphomas on distinct endocytic processes and how these alterations may affect cell signaling holds promise for uncovering novel disease mechanisms and identifying therapeutic targets, particularly in the context of advancing precision medicine.

The heterogeneity observed in CD79b expression within our data may carry significant clinical implications for anti-CD79b antibody–drug conjugate (ADC) therapies. Specifically, anti-CD79b monoclonal antibodies (mAbs), such as clone SN8, have demonstrated binding capabilities and efficient internalization into lymphoma cells, forming the rationale for developing ADCs as a treatment strategy for non-Hodgkin lymphomas [29,30].

Contrasting findings emerge from studies investigating correlations between CD79b expression and the efficacy of CD79b-ADCs. Dornan et al. reported a substantial dynamic range in CD79b expression on the cell surface of DLBCL cell lines, identifying a threshold effect where cases below a specific level of antigen expression exhibited insensitivity to the maximum amount of anti-CD79b-vcMMAE tested [13]. In contrast, Pfeifer et al. observed that the response to anti-CD79b MMAE did not correlate with antigen expression on DLBCL cell lines, suggesting that a specific cut-off level of CD79b expression may not be a prerequisite for the efficacy of PV [6]. Similarly, in a DLBCL preclinical model, BJAB and Granta xenograft tumors responded to anti-CD79b despite having lower surface expression levels of CD79b compared to normal B cells, and the efficacy of anti-CD79b ADCs did not align with surface expression levels of CD79b [31]. In a phase 1b-2 study evaluating the safety and activity of PV in combination with R-CHP in previously untreated DLBCL pts, Tilly et al. did not find an association between CD79b expression levels evaluated with an IHC score and treatment response or PFS [7]. These preclinical data, coupled with limited clinical experience, cast doubt on whether CD79b expression levels are a major determinant of the response to CD79b-ADC. Instead, the influence of the COO on the efficacy of regimens containing the CD79b-ADC polatuzumab-vedotin (PV) for DLBCL is well established. A recent analysis considering data from clinical trials involving pts with relapsed or refractory disease and corroborated by data from the POLARIX trial provided compelling evidence that the ABC subtype is significantly more responsive than GCB to schemes containing PV [32]. This observation is consistent with preclinical studies implicating CD79b-dependent vulnerability of ABC DLBCLs [33].

While our study did not assess CD79b mutations, it is crucial to acknowledge the impact of hotspot mutations within the intracytoplasmic immunoreceptor tyrosine-based activation motif (ITAM) of CD79b, particularly identified in ABC DLBCLs, on the level of CD79b surface expression. Indeed, several of the mutated variants play a pivotal role in “chronic active” BCR signaling, increasing surface expression of CD79b/Ig on B-cells, inhibiting BCR internalization, enhancing BCR clustering, and attenuating LYN kinase, a feedback inhibitor of BCR signaling. In particular, it has been demonstrated that lymphoma levels of CD79b are strongly correlated with crosslinking BCR-induced signaling [33]. In coherence with this observation, it has been noted that the presence of CD79b mutations does not impair the response to anti-CD79b-MMAE in vitro models [6]. Nevertheless, it is noteworthy that we observed higher CD79b surface expression in pts aged over 60 years and those with high-risk disease (R-IPI 3-5), factors associated with a greater benefit from the addition of the CD79b-ADC PV to standard first-line chemotherapy in the phase III Polarix study [15]. We do not think that age directly influences CD79b expression but that the biology of DLBCL in the elderly differs from DLBCL in younger pts; in particular, the incidence of DLBCL of the non-GC B-cell type is known to increase with age. Our findings offer indirect evidence that this benefit may indeed be linked to higher CD79b surface expression in pts with these specific characteristics, suggesting a potential avenue for tailoring treatment strategies based on CD79b expression profiles.

While obtaining tissue biopsies for flow cytometric (FC) analysis is not typically part of routine diagnostic work-up for DLBCL, we believe that quantitative FC could provide valuable insights into target antigens like CD79b. The clinical significance of CD79b extends beyond CD79b-ADC therapies, with its potential as a target for CAR-T cell therapy in B-cell lymphomas gaining interest [34,35]. Future studies should investigate whether quantitative FC analysis of CD79b expression in DLBCL can provide predictive information for the response to first-line therapy incorporating CD79b-ADC PV. Additionally, we also think that future research should focus on gathering longitudinal data from newly diagnosed and relapsed cases to investigate variations in CD79b surface expression throughout treatment.

## 5. Conclusions

In conclusion, our observations regarding the heterogeneous surface expression of CD79b correlating with the intensity of the light chain expression in aggressive B-cell lymphomas align well with our existing comprehension of the role of the BCR complex in this particular subtype of lymphomas [36].

## Figures and Tables

**Table 1:** Patients’ characteristics.

Characteristics	All Patients (127)	Diagnosis (101)	Relapse (26)
**AGE**	65 yrs (range 19–88)	64 yrs (ranges 22–88)	69 yrs (range 19–83)
**SEX**	63 M/64 F	50 M/51 F	13 M/13 F
**ECOG PS**			
0	46 (36%)	36 (35%)	10 (38.5%)
1	45 (35%)	34 (34%)	11(42%)
2	17 (14%)	15 (15%)	2 (8%)
3	13 (10%)	10 (10%)	3 (11.5%)
4	6 (5%)	6 (6%)	0 (0%)
**HISTOTYPE**			
High-grade lymphoma	13	10	3
t-FL	11	6	5
NOS, non-GC (Hans’ algorithm)	48	37	11
NOS, GC (Hans’ algorithm)	33	31	2
PMLBCL	12	12	0
Other/not classifiable	10	5	5
**STAGE**			
1	2	2	0
2	30	25	5
3	27	22	5
4	68	52	16
**IPI**			
0	10	9	1
1	20	17	3
2	28	20	8
3	30	23	7
4	21	17	4
5	18	15	3
**R-IPI**			
0	10	9	1
1–2	48	37	11
3–5	69	55	14
**TREATMENT (1st LINE)**			
R-CHOP/R-COMP	77		
R-miniCOMP	15		
R-CVP/others	9		
**CD79b%**			
<1%	23 (18%)	18 (18%)	5 (19%)
1–19%	11 (9%)	6	5
20–69%	25 (20%)	21	4
≥70%	68 (53%)	56	12

## Data Availability

The data presented in this study are available in this article.
